# Management of Insomnia Complaints by Non‐Sleep Specialist Physicians: A French DELPHI Consensus

**DOI:** 10.1111/jsr.70143

**Published:** 2025-07-17

**Authors:** Pierre‐Alexis Geoffroy, Jean‐Louis Pépin, Marjorie Guillet, Jean‐Bastien Micoulaud‐Franchi, Yves Dauvilliers, Damien Léger, Sylvie Royant‐Parola

**Affiliations:** ^1^ Department of Psychiatry and Addictology AP‐HP, GHU Paris Nord, DMU Neurosciences, Bichat ‐ Claude Bernard Hospital Paris France; ^2^ ChronoS Center GHU Paris – Psychiatry & Neurosciences Paris France; ^3^ Université Paris Cité, Inserm, NeuroDiderot Paris France; ^4^ Grenoble Alpes University, HP2 Laboratory Grenoble France; ^5^ Dr. Marjorie Guillet's Practice France; ^6^ Arsac Health Center Arsac France; ^7^ National Reference Center for Narcolepsy and Rare Hypersomnias, Sleep and Wakefulness Disorders Unit Gui‐de‐Chauliac Hospital, Montpellier, Institute of Neurosciences of Montpellier (INM), Inserm, University of Montpellier Montpellier France; ^8^ INSERM, Institute of Neurosciences of Montpellier Montpellier France; ^9^ AP‐HP, Hôtel‐Dieu Centre du Sommeil et de la Vigilance France; ^10^ Université Paris Cité, VIFASOM (Vigilance, Fatigue, Sleep, and Public Health), UMR Paris France; ^11^ Réseau Morphée Garches France

**Keywords:** clinical guidelines, healthcare providers, multidisciplinary steering committee, primary care approach, sleep management

## Abstract

Insomnia is the most prevalent sleep disorder and a major public health concern, affecting chronically up to 19% of the adult population in France. Despite its significant impact on quality of life, mental health, and cardiometabolic disease, insomnia disorder remains underdiagnosed and inadequately managed. General Practitioners (GPs) play a pivotal role in addressing insomnia complaints but often face constraints, such as limited consultation time and a lack of specialised training. To provide practical recommendations for non‐sleep specialist physicians, a French Delphi consensus was conducted based on recent expert guidelines. A multidisciplinary Steering Committee formulated 21 clinical statements regarding the evaluation, severity assessment, management, and referral to sleep specialists of patients presenting with insomnia disorder. These statements were submitted to a panel of 37 GPs across France via two rounds of anonymous rating using a 9‐point Likert scale. While a consensus was reached for 95% of the statements, encompassing initial evaluation, sleep hygiene recommendations, behavioural interventions, and referral criteria, controversies emerged regarding the prescription of hypnotics, underscoring areas requiring further clarification and education. Our results highlight the importance of tailored approaches in primary care, emphasising pragmatic strategies rather than highly specialised protocols. This French Delphi consensus provides a structured yet flexible framework to support insomnia disorder management by non‐sleep specialist physicians, bridging the gap between guidelines and real‐world clinical practice.

## 1 Introduction

Insomnia disorder affects approximately 10 to 19% of the adult population in its chronic form (Chan‐Chee et al. 2011; Leger et al. 2000; Morin and Jarrin 2022; Riemann et al. 2022). Its impact on health is significant, with long‐term cardiometabolic consequences and increased cardiovascular mortality (Hong et al. 2025; Zheng et al. 2019). Thus, insomnia disorder affects quality of life and contributes to marked distress in many patients, prompting them to seek medical care (Morin and Jarrin 2013). However, insomnia remains largely underdiagnosed and inadequately managed, with unmet needs in its management (Campbell et al. 2023; O'Regan et al. 2023).

Insomnia disorder presents a heterogeneous clinical profile, encompassing distinct phenotypes. These include sleep‐onset insomnia (SOI: difficulty initiating sleep), sleep‐maintenance insomnia (SMI: frequent or prolonged nocturnal awakenings), late or terminal insomnia—also termed early‐morning awakening (EMA: premature awakening with an inability to return to sleep), and mixed insomnia, which combines two or more of these complaints. Less common but clinically relevant presentations comprise paradoxical insomnia, in which objective sleep recordings are normal despite the patient's perception of sleeplessness, and psychophysiological insomnia with conditioned hyper‐arousal, characterised by learned associations between the sleep environment and heightened cognitive or somatic activation (Coelho et al. 2023; Fietze et al. 2021). In the cohort of 456 patients reported by Fietze et al. 54% presented with SOI, 66% with SMI, 45% with EMA, and 57% with a mixed profile, making maintenance difficulties the most frequent complaint (Fietze et al. 2021). These subtypes highlight the need for a precise clinical evaluation to tailor a personalised intervention.

Insomnia disorder is associated with an increased risk of psychiatric comorbidities, such as anxiety and depression (Baglioni et al. 2011; Basquin et al. 2024; Pandi‐Perumal et al. 2020; Solelhac et al. 2024), as well as neurological (De Bergeyck and Geoffroy 2023; Mayer et al. 2011). Moreover, there is substantial evidence that insomnia disorder is a prevalent residual symptom, even after successful treatment of depression (Higson‐Sweeney et al. 2024), and that its persistence increases the risk of relapse (Geoffroy et al. 2021; Perlis et al. 1997).

In France, general practitioners (GPs) diagnosed and prescribed treatment for more than 60% of patients suffering from chronic insomnia disorder (Chalet et al. 2024; SFTG—HAS 2007). These frontline physicians therefore constitute one of the key primary healthcare resources for insomnia management (Morin et al. 2006). However, GPs do not systematically receive specific training in sleep medicine. Insomnia is often addressed at the end of an appointment or during prescription renewals, while GPs face limited consultation time. This may also hinder the implementation of management recommendations (Edinger and Sampson 2003; Léger et al. 2005; Robert et al. 2019).

European guidelines on the diagnosis and management of adult insomnia disorder have recently been updated and are particularly relevant for healthcare professionals trained in sleep medicine (Riemann et al. 2023). Whilst they should also be useful for GPs and other specialists, their implementation may be limited as they do not consider the specific constraints of frontline medical practice. In this context, it remains unclear whether non‐specialist physicians in sleep and insomnia are aware of these guidelines and integrate them into their clinical practice. In France, the most recent national recommendations for the treatment of insomnia were issued jointly by the SFTG (Society for Therapeutic Training of General Practitioners) and HAS (French National Authority for Health) in 2006 (SFTG—HAS 2007). However, these guidelines have not been updated at the time of this study, and no international guidelines specifically developed for non‐sleep specialists currently exist. This reinforces the need for updated context‐specific guidance that addresses the real‐life constraints of general practice.

Given these challenges, the development of specific recommendations for GPs who serve as the first line of healthcare in France appears beneficial to improve insomnia management. A Delphi‐type consensus approach involving multidisciplinary experts was implemented in France. It brought together sleep specialists and GPs to propose consensus statements for practical clinical recommendations adapted for the management of insomnia by frontline physicians. In addition to establishing consensus recommendations, this study also aimed to identify areas of clinical uncertainty and practical barriers encountered in routine insomnia care, to inform future training and support strategies for general practitioners.

## 2 Methods

The Delphi methodology has been used in various therapeutic fields, including sleep medicine (Geoffroy et al. 2023; Ohayon et al. 2017, 2018; Steier et al. 2023). This methodology is an iterative approach used to derive a consensus based on information collected from a panel of participants with expertise in the considered topic (Diamond et al. 2014; HAS, Haute Autorité de Santé 2010; Hsu and Sandford 2007; Skinner et al. 2015). Invited participants provide individually and anonymously their level of agreement on statements formulated by a Steering Committee (SC) to reach a consensus on a defined subject.

In accordance with the French National Health Authority and international recommendations, the voting group indicated their level of agreement with statements using a 9‐point Likert scale ranging from 1 “*Strongly disagree*” to 9 “*Strongly agree*”, during two voting rounds (Appendix S1); (Diamond et al. 2014; HAS, Haute Autorité de Santé 2010; Skinner et al. 2015). The percentage of scores and the median were calculated separately for each statement in each voting round. A *strong consensus* was achieved for a statement when more than 75% of the scores were ≥ 7 and the median score was ≥ 8. When only one of these two criteria was met, the statement was considered to have reached a *good consensus* (Diamond et al. 2014; McMillan et al. 2016; Skinner et al. 2015). The current study took place from April 2023 to September 2024.

### 2.1 Steering Committee (SC) and Statements

The SC consisted of seven experts with five sleep medicine specialists, including two psychiatrists, one neurologist, one pulmonologist, and one occupational health physician, as well as two frontline GPs.

Based on existing recommendations (Chesson et al. 1999; Riemann et al. 2023, 2017; Sateia et al. 2017; Schutte‐Rodin et al. 2008) and their clinical experience, SC members formulated 20 statements regarding the diagnosis, severity, management, and referral of patients presenting with insomnia complaints. These statements were then reviewed by a committee of five patients with chronic insomnia to ensure these statements took the perspective of their experience into consideration. These patients were suggested by members of the SC. All had been diagnosed with insomnia disorder and were receiving follow‐up care in one of the sleep center where the co‐authors currently work.

After a second voting round, a supplementary statement was formulated by the SC which led to a total number of statements of 21. [Correction added on 29 August 2025, after first online publication: The preceeding sentence has been corrected in this version.]

### 2.2 Composition of the Voting Group

To ensure broad national representation, experts for the voting group were recruited during the French General Medicine Congress (CMGF) in March 2024. GPs had the opportunity to participate in this study and were selected based on several criteria, that is, involvement and experience in managing patients with sleep complaints, years of practice with a mix of very experienced and less experienced GPs, diversity in geography (Appendices S2 and S3), and representation of different practice settings (that is, private practice, mixed, and hospital‐based practice). Involvement and experience in managing sleep complaints were assessed based on self‐reported regular clinical exposure to patients with insomnia or other sleep‐related symptoms (that is, average number of spontaneous insomnia complaints per week of consultations and average number of insomnia complaints expressed after physician inquiry).

Eighty‐seven physicians expressed interest at the event and were subsequently invited by email to participate in online voting, with personalised access through a dedicated website. Participants remained anonymous throughout the process and had no interaction with the SC, whose members did not participate in the voting in accordance with methodological recommendations (HAS, Haute Autorité de Santé 2010). A quota of 30 respondents per statement was set to ensure robustness in the reached consensus (HAS, Haute Autorité de Santé 2010; Letrilliart et al. 2009; McMillan et al. 2016).

### 2.3 Conduct of the Voting Rounds

For the first voting round, 87 participants were invited to participate and 37 provided their opinion on the statements formulated by the SC, with the option to leave comments, allowing them to elaborate on their ratings. At the end of this first round, participants' scores and comments were summarised for each item and reviewed by the SC:

- Statements that reached a *strong consensus* were validated with no changes and included in the final synthesis.
- Statements that obtained a *good consensus* or *did not reach a consensus* were revised by the SC based on voters' comments and submitted for a second voting round.

The 37 GPs who participated in the first voting round were invited to the second round of the online questionnaire incorporating the revised statements. The option to leave comments was replaced by a “No Opinion” option, to allow panellists to abstain from rating revised statements they did not feel comfortable with. In line with the Delphi methodology, these responses were excluded from the consensus analysis to ensure that statistical analyses captured only informed expert judgements, thereby enhancing the validity of consensus measurement and minimising noise from non‐informative responses.

### 2.4 Ethical Considerations

This study was conducted in accordance with the Declaration of Helsinki. Personal data collected during this study were anonymised and dissociated from the results, in compliance with French data protection laws (GDPR—General Data Protection Regulation).

## 3 Results

A total of 21 statements formulated by the SC was evaluated by a panel of GPs (Appendices S2 and S3). Out of the 37 GPs who participated in the first voting round, 31 responded to the second round, corresponding to an attrition rate of 16% between the two rounds.

For each topic, the distribution of votes, medians, and results are presented in Tables 1, 2, 3, 4, 5, 6. After the first voting round, 12/20 statements achieved a *strong consensus*; 4/20 statements reached a *good consensus*, and 4 statements did not reach a consensus. The SC, therefore, revised eight statements for the second voting round. Among them, a statement concerning insomnia coupled with clinically significant depressive and/or anxiety symptoms, deemed important by the SC members, was reworded into two distinct statements (Nos. 17 and 18, Tables 4 and 5, respectively). Nine statements were submitted for the second voting round, following which 6/9 statements achieved a *strong consensus*, 2/9 reached a *good consensus*, and 1 statement did not reach a consensus.

**TABLE 1:** Statements related to the initial assessment of insomnia and early interventions.

Statements		Votes (*n*)	Values 1–3 (*n*)	Values 4–6 (*n*)	Values 7–9 (*n*)	Median	Results
1	In response to an insomnia complaint, even if consultation time is limited, an initial assessment of insomnia severity should be performed: Insomnia as a reaction to a difficult life event vs. chronic/long‐standing insomnia Frequency of nights with less than 5 h of sleep Symptom severity and perceived impairment Potential risks of daytime consequences	37	0% (0)	16% (6)	84% (31)	8	Strong consensus
2	In response to an insomnia complaint, even if consultation time is limited, significant distress justifying immediate intervention should be assessed (e.g., depression with suicide risk).	37	0% (0)	13% (5)	87% (32)	9	Strong consensus
3	In response to an insomnia complaint, even if consultation time is limited, a potential underlying or associated comorbidity should be identified, whether iatrogenic (e.g., recent treatment for a somatic or psychiatric condition) or medical (e.g., pain, anxiety, mood disorder).	37	0% (0)	8% (3)	92% (34)	8	Strong consensus
4	If insomnia is recent or mild, patient should be reminded of sleep hygiene rules and asked to report any initial results from behavioural changes at their next consultation.	37	5% (2)	17% (6)	78% (29)	9	Strong consensus

[Correction added on 29 August 2025, after first online publication: In this table, Statement 4 has been corrected in this version.]

**TABLE 2:** Statements related to long‐standing or severe cases, and insomnia assessment tools.

Statements		Votes (*n*)	Values 1–3 (*n*)	Values 4–6 (*n*)	Values 7–9 (*n*)	Median	Results
5	If insomnia is long‐standing or severe, it requires time and often multiple consultations for an accurate assessment and to determine potential underlying causes or comorbidities, in order to define the best management approach. Therefore, if time is lacking, a follow‐up appointment should be scheduled within the next few weeks.	37	0% (0)	14% (5)	86% (32)	9	Strong consensus
6	If insomnia is long‐standing or severe, it may be useful to provide the patient with a sleep assessment questionnaire of your choice* to be completed and brought back to the next consultation, allowing for time efficiency and guidance in management. *For example: www.questionnairedusommeil.fr	31	0% (0)	6% (2)	94% (29)	8	Strong consensus
7	If insomnia is long‐standing or severe, the patient should be given a sleep diary—essential for an accurate assessment of their disorder— to be completed and brought back to the next consultation.	37	3% (1)	16% (6)	81% (30)	8	Strong consensus
8	If time allows or during the next consultation, if the sleep diary has not been completed, sleep disorders should be assessed by gathering the following information: • Bedtime and wake‐up time: average, regularity; weekdays vs. weekends • Sleep onset latency • Number of nighttime awakenings • Duration of nighttime awakenings • Total sleep duration per night • Time spent in bed without sleeping and activities during these wake periods • Sleep debt (difference between perceived sleep duration and desired sleep duration) • Nap time and duration	37	8% (3)	14% (5)	78% (29)	9	Strong consensus

*Note*: For each statement, a total number of respondents different from 37 or 31 indicates the presence of “No Opinion” responses.

**TABLE 3:** Statements related to comorbidities, lifestyle habits, and daytime consequences of insomnia.

Statements		Votes (*n*)	Values 1–3 (*n*)	Values 4–6 (*n*)	Values 7–9 (*n*)	Median	Results
9	If time allows or during the next consultation, potential somatic or psychiatric comorbidities should be investigated, such as: • Depressive symptoms, anxiety symptoms • Sleep apnea (Obstructive Sleep Apnea Syndrome – OSAS), Restless Legs Syndrome (RLS) • Chronic disease: neurological, pulmonary, metabolic, etc. • Pain …and the onset date of insomnia should be compared with the appearance of these conditions or their treatments.	37	0% (0)	0% (0)	100% (37)	9	Strong consensus
10	If time allows or during the next consultation, the patient's lifestyle habits should be assessed to identify one or more potentially modifiable causes: • Sleep environment: partner, darkness, noise, etc. • Work and leisure schedules/rhythms • Weekday vs. weekend/holiday sleep patterns • Pre‐sleep activities • Physical activity (absent, occasional, regular) • Stimulant consumption, medication dependence • Screen use before bedtime	37	0% (0)	14% (5)	86% (32)	9	Strong consensus
11	If time allows or during the next consultation, the potential daytime consequences of insomnia, which are severity factors to consider, should be evaluated: • Daytime sleepiness, fatigue, attention deficits • Limitations in personal and professional life • Risk of accidents • Irritability/stress	31	4% (1)	0% (0)	96% (30)	9	Strong consensus

*Note*: For each statement, a total number of respondents different from 37 or 31 indicates the presence of “No Opinion” responses.

**TABLE 4:** Statements related to first‐line management (pharmacological and behavioural approach).

Statements		Votes (*n*)	Values 1–3 (*n*)	Values 4–6 (*n*)	Values 7–9 (*n*)	Median	Results
12	Since severe insomnia is often a fluctuating and multifactorial condition, the patient should be informed that: • Medication alone is rarely sufficient • Non‐pharmacological (behavioural) management can be effective for certain insomnia profiles • Relaxation or sophrology techniques are potentially useful only in cases of sleep onset difficulties and most often in combination with another treatment approach.	37	8% (3)	13% (5)	79% (29)	9	Strong consensus
13	It is advisable to prescribe hypnotics for a short duration in the following cases: ○ Major sleep debt unbearable for the patient ○ Potentially dangerous daytime consequences of insomnia ○ Acute stress following a traumatic life event ○ Depression with insomnia and suicidal crisis	30	10% (3)	17% (5)	73% (22)	7.5	No consensus
14	The use of hypnotics should be temporary and/or occasional. Indeed, there are real risks of dependence and/or adverse effects in cases of prolonged use.	37	0% (0)	0% (0)	100% (37)	9	Strong consensus
15	Patients should be reassured about the benefit/risk balance of short‐term and temporary use of hypnotics.	30	4% (1)	20% (6)	76% (23)	8.5	Strong consensus
16	Based on the sleep diary assessment—and after ruling out diagnoses such as Restless Legs Syndrome, sleep apnea, depression, anxiety, or pain, which require prior specific management—a behavioural approach such as Cognitive Behavioural Therapy for Insomnia (CBT‐I) can be proposed to the patient.	37	2% (1)	22% (8)	76% (28)	8	Strong consensus
17	In cases of insomnia associated with depressive and/or anxiety disorders, both conditions should be treated simultaneously.	31	4% (1)	22% (7)	74% (22)	8	Good consensus

*Note*: For each statement, a total number of respondents different from 37 or 31 indicates the presence of “No Opinion” responses.

**TABLE 5:** Statements related to patient referral.

Statements		Votes (*n*)	Values 1–3 (*n*)	Values 4–6 (*n*)	Values 7–9 (*n*)	Median	Results
18	In cases of insomnia associated with depressive and/or severe anxiety disorders that are resistant to well‐conducted initial management in general practice, a psychiatric consultation should be sought.	31	5% (1)	15% (5)	80% (25)	8	Strong consensus
19	Apart from associated comorbidities, referring the patient to a sleep specialist (hospital‐based or private practice) is appropriate in the following situations: ○ Long‐standing insomnia ○ Refractory insomnia ○ Suspected sleep apnea syndrome or Restless Legs Syndrome ○ Severe insomnia with very short sleep duration	30	7% (2)	3% (1)	90% (27)	9	Strong consensus

**TABLE 6:** Statements related to the use of polysomnography.

Statements		Votes (*n*)	Values 1–3 (*n*)	Values 4–6 (*n*)	Values 7–9 (*n*)	Median	Results
20	Polysomnography should be analysed by any sleep specialist, whether in a hospital or private practice setting.	28	0% (0)	14% (4)	86% (24)	8	Strong consensus
21	In cases of chronic insomnia and suspected sleep apnea syndrome, the gold standard examination is polysomnography rather than ventilatory polygraphy.	25	16% (4)	12% (3)	72% (18)	8	Good consensus

*Note*: For each statement, a total number of respondents different from 37 or 31 indicates the presence of “No Opinion” responses.

Overall, 18/21 statements (86%) achieved a *strong consensus*, and 2/21 statements (9%) reached a *good consensus*, resulting in a consensus rate of 95%.

### 3.1 Initial Insomnia Assessment and Early Interventions (Table 1)

Four statements focused on the initial assessment of insomnia, applicable within a time‐limited consultation setting. All were validated with a *strong consensus* after the first voting round. Voters acknowledged the necessity of conducting an initial assessment of insomnia severity when a patient presents with this complaint, as well as identifying significant distress that may justify immediate management (statements 1 and 2). They also endorsed the identification of potential underlying causes or comorbidities, along with reinforcing sleep hygiene principles for patients (statements 3 and 4).

### 3.2 Severe Cases and Assessment Tools (Table 2)

The four statements relating to cases of long‐standing or severe insomnia were all validated with a *strong consensus*. According to the panel, it is important to take time and schedule multiple consultations to accurately assess insomnia. The use of various tools provided to patients, such as questionnaires or a sleep diary, can help save time in future consultations and improve insomnia phenotyping (statements 6 and 7). If these tools have not been used by the patient, voters agreed on conducting a detailed assessment of their sleep habits during the next appointment.

### 3.3 Comorbidities, Lifestyle Habits, and Daytime Consequences of Insomnia (Table 3)

Three statements related to comorbidities, lifestyle habits, and the daytime consequences of insomnia were proposed. All of these statements were validated with a *strong consensus*.

Voters agreed with the proposed recommendation to investigate potential somatic or psychiatric comorbidities and to consider the chronology of symptoms or the initiation of treatment.

The panel also agreed that exploring the patient's lifestyle habits and identifying potentially modifiable factors was important (statement 10) and that the daytime consequences of insomnia should also be considered (statement 11).

### 3.4 First‐Line Management (Behavioural and Pharmacological Approaches) (Table 4)

The SC formulated six statements regarding first‐line management by non‐sleep specialist physicians. Four received a *strong consensus* and were validated; one, statement No. 17, related to insomnia associated with depressive and/or anxiety disorders, achieved a *good consensus*. However, statement No. 13, concerning the prescription conditions of hypnotics, did not reach a consensus.

The panel of voters agreed that insomnia is a fluctuating and multifactorial condition requiring specific communication with the patient regarding its therapeutic and behavioural management.

Statement No. 13, which recommended short‐term hypnotic prescriptions in certain specific cases (major unbearable sleep debt for the patient; potentially dangerous daytime consequences of insomnia; acute stress following a traumatic life event; depression with insomnia and suicidal crisis), was not approved by the panellists. They reached a strong consensus that the risk of dependence and adverse effects requires only temporary use (statement 14) and should be preceded by patient reassurance (statement 15). The term “*hypnotics*” refers here to benzodiazepine receptor agonists, including benzodiazepines and Z‐drugs, and other sedative agents—such as first‐generation antihistamines and low‐dose sedating antidepressants.

After performing a differential diagnosis justifying a specific treatment, the panel recommended offering a cognitive and behavioural approach, such as Cognitive Behavioural Therapy for Insomnia (CBT‐I), to patients suffering from chronic insomnia.

In cases of insomnia with severe depressive and/or anxiety disorders, voters advocated for the simultaneous treatment of both conditions (s*tatement 17*).

### 3.5 Patient Referral (Table 5)

Two statements regarding the referral of patients to a sleep specialist were submitted for voting by the panel and were validated with a *strong consensus*.

Voters agreed with the recommendation to seek a psychiatrist's opinion in cases of insomnia associated with depressive and/or severe anxiety disorders that are resistant to initial management (statement 18).

The panel considered that referral to a sleep specialist is necessary in the following situations: long‐standing or refractory insomnia, suspected sleep apnoea syndrome or Restless Legs Syndrome, and severe insomnia with very short sleep duration.

### 3.6 Use of Polysomnography (Table 6)

The use of polysomnography in the assessment of insomnia was addressed in two statements, which were validated with a *strong consensus* (statement No. 20) and a *good consensus* (statement No. 21).

The panellists recommended that polysomnography should be analysed by any sleep specialist. According to the voting experts, this examination remains the gold standard in cases of chronic insomnia and suspected sleep apnoea syndrome.

## 4 Discussion

**FIGURE 1:**
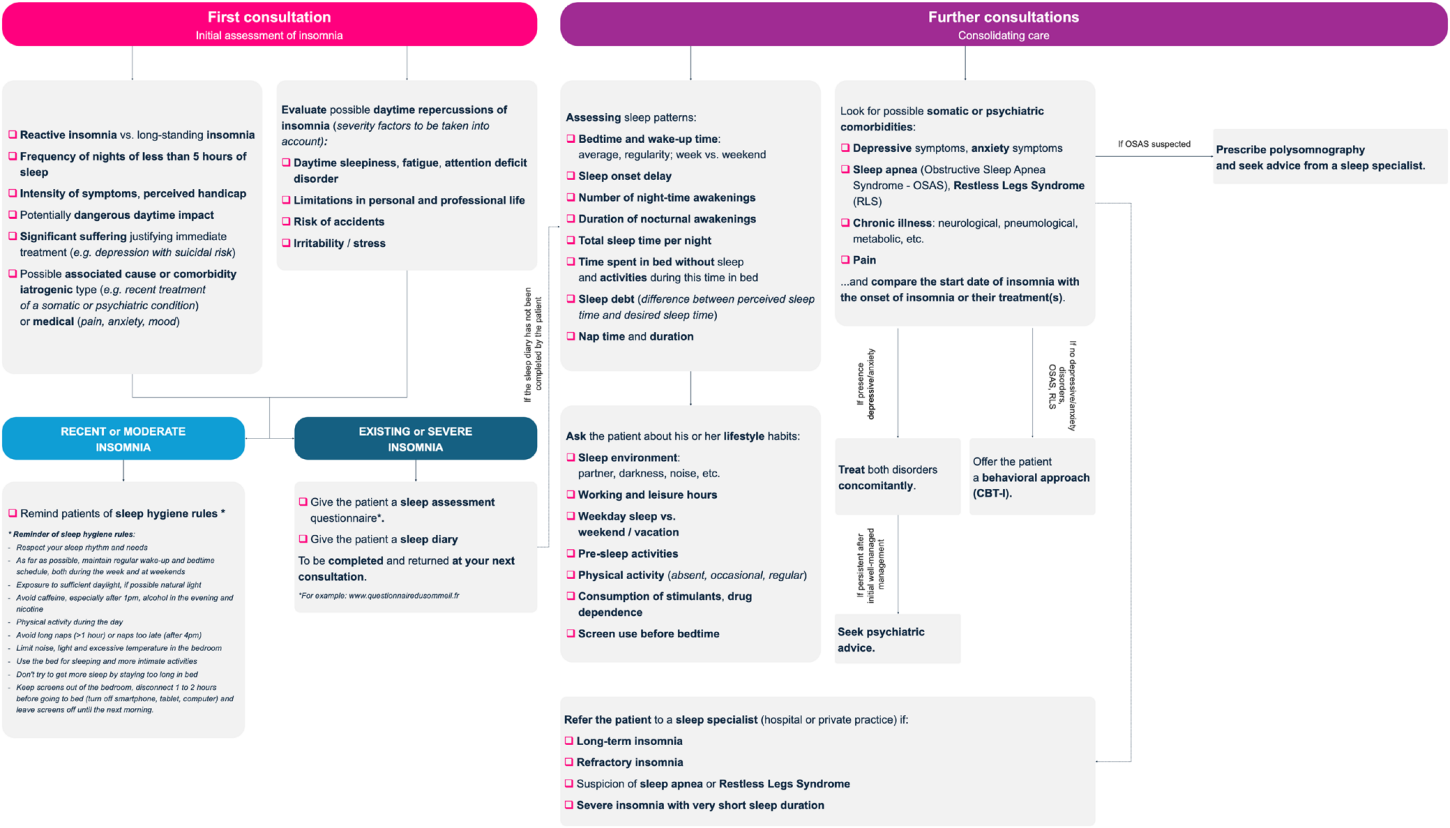
Insomnia complaints management algorithm in general practice.

The main objective of this study was to develop a consensus on the management of insomnia complaints by non‐sleep specialist physicians. Through the Delphi method, a multidisciplinary expert group was able to establish concrete and practical recommendations tailored to the clinical practice of frontline physicians (Figure 1). Of the 21 statements formulated by the SC, 20 reached a consensus, resulting in a 95% validation rate by the panellists.

Overall, the results obtained regarding statements concerning the initial assessment of insomnia complaints, assessment tools, and the consideration of comorbidities align with the routine practice of frontline physicians. They highlight essential and specific elements for GPs that had not been previously established. Indeed, the recommendations developed through this national consensus complement the recently published European guidelines (Riemann et al. 2023). Although these guidelines are detailed—covering a wide range of aspects related to insomnia diagnosis and treatment—and intended for all practitioners involved in insomnia disorder management, they do not consider the specific time constraints frontline physicians are confronted with, as well as their heterogeneous backgrounds and knowledge on sleep and sleep disorders. As a result, they are not readily applicable in routine primary care practice. The statements derived from our consensus offer a more pragmatic approach adapted to real‐world practice.

Although sleep diaries and detailed questionnaires were underscored in this consensus as useful tools for phenotyping insomnia and guiding management, other ultra‐brief screening tools may offer a pragmatic alternative in time‐constrained consultations. The 2‐item (ISI‐2) and 3‐item (ISI‐3) versions of the Insomnia Severity Index have been validated and are increasingly used for the rapid assessment of insomnia severity or monitoring changes over time (Kraepelien et al. 2021; Thakral et al. 2021). These short assessment tools can facilitate early identification of insomnia symptoms and support follow‐up in time‐limited clinical contexts.

Despite achieving a high consensus rate for nearly all statements issued by the SC, some statements received more heterogeneous responses from the panel, reflecting the existence of unmet needs. One of the debated topics concerned the use of hypnotics, particularly their prescription in specific cases. Although the median vote reached 7.5, and 73% of voters expressed a favourable trend toward short‐term prescribing, the lack of consensus indicates a continued need to clarify this issue. This statement was likely rejected by the voting group due to overly broad and mixed conditions (e.g., depression with sleep deprivation, drowsiness, post‐traumatic stress disorder), as well as the term “hypnotics,” which may have been considered too vague for non‐specialist practitioners. These results were somewhat anticipated by the SC in light of recent data linking hypnotic use to an increased risk of suicide attempts and suicide (Tournier et al. 2023), despite other interventional studies showing rather a protective effect (Maruani et al. 2023; McCall et al. 2019). Nevertheless, public awareness of this issue may have heightened prescribers' concerns, leading them to restrict prescriptions of these treatments. However, some interventional studies have shown that hypnotic use is associated with a reduced risk of suicidal ideation (Maruani et al. 2023), a finding observed in clinical practice by SC members. This Delphi consensus emphasises a stepwise use of hypnotic agents in primary care. According to the 2023 European insomnia guideline (Riemann et al. 2023), pharmacotherapy should be limited to short courses (≤ 4 weeks) when cognitive‐behavioural therapy for insomnia (CBT‐I) is unavailable, contraindicated, or insufficient. Evidence‐based first‐line options encompass benzodiazepines, benzodiazepine‐receptor agonists or “Z‐drugs”, dual orexin‐receptor antagonists, and very‐low‐dose sedating antidepressants. In adults aged ≥ 55 years, prolonged‐release melatonin 2 mg—presently the only melatonin‐receptor agonist licensed in Europe—may be administered for up to 13 weeks and is favoured when conventional hypnotics are poorly tolerated. Should chronic insomnia persist despite optimal behavioural care, longer‐term use of agents with the most favourable safety and dependence profiles may be considered under stringent clinical supervision with predefined re‐evaluation intervals. Irrespective of the molecule selected, pharmacotherapy must remain integrated within a multimodal strategy that prioritises CBT‐I, structured sleep‐hygiene counselling, and longitudinal monitoring of efficacy and adverse effects.

The semiological complexity of insomnia deserves particular attention in clinical practice. While our consensus recognises its fluctuating and multifactorial nature, the heterogeneity of its presentations—ranging from sleep‐onset and sleep‐maintenance insomnia to terminal insomnia, paradoxical insomnia, and conditioned hyperarousal—has important implications for decision‐making. Each phenotype may reflect distinct pathophysiological mechanisms, requiring individualised therapeutic approaches. Recent work on precision semiology supports this perspective, advocating for a more nuanced characterisation of insomnia to align interventions to patient profiles (Coelho et al. 2023). Integrating such an approach into primary care could improve diagnostic accuracy, facilitate the distinction between transient complaints and chronic insomnia disorder, and support more personalised and efficient care—particularly in time‐constrained settings.

As this study was conducted in France, it is also important to note that French GPs receive financial incentives from the National Health Insurance Fund (*Rémunération sur objectifs de santé publique*—ROSP), in which the prescription of benzodiazepine‐type hypnotics appears in 2 out of 15 performance indicators (Caisse Nationale de l'Assurance Maladie (CNAM) 2024). It involves granting additional compensation to physicians who agree to adapt their practices based on specific criteria, both medical and economic. This mechanism may influence the prescription of this therapeutic class in France. This debate underscores the importance of continuously reminding prescribers about the safety and tolerability profile of these treatments to help them weigh the benefit–risk balance of available medications based on their patients' profiles (Yue et al. 2023).

Two other statements, although receiving good consensus, sparked discussions within the panel during the voting rounds and the SC during the analysis of responses. The first, concerning the management of insomnia associated with anxiety and/or depressive disorders, was close to reaching a strong consensus, with a median vote of 8% and 74% of participants in agreement with this recommendation. These results indicate that this management approach is not yet fully optimised. It would have been useful to specify that, in the absence of urgency, a stepwise approach could be considered: initiating a first treatment, ensuring regular follow‐up, and then reassessing the need to introduce a second treatment concurrently to manage both conditions simultaneously. The second statement, with a median vote of 8 and agreement from 72% of voters, aimed to remind practitioners of the gold standard examination for insomnia associated with suspected sleep apnea syndrome. The fact that this recommendation did not reach a *strong consensus* suggests that some knowledge gaps persist among frontline physicians regarding the respective indications for polysomnography and ventilatory polygraphy, or even some confusion between these two tests. Additionally, accessibility constraints may explain why GPs tend to prescribe more frequently ventilatory polygraphy, as it appears more readily available in outpatient settings than polysomnography. It is therefore essential to continue raising awareness among healthcare professionals about these issues. Although these two statements reached only a *good consensus* rather than a *strong one*, they were retained given their clinical relevance and proximity to the *strong consensus* threshold (statements 17 and 21 each reached a median score of 8, with 74% and 72% of respondents, respectively, rating them ≥ 7—just below the 75% threshold defined for *strong consensus*). These statements addressed key issues frequently encountered in general practice—namely, the management of insomnia in the context of comorbid anxiety or depressive disorders and the indication of polysomnography for suspected sleep apnea. Both were considered essential by the SC, as they reflect situations with significant impact on care and are supported by current clinical evidence and guideline recommendations.

Although the Delphi method is a well‐recognised and structured procedure, it has certain limitations related to the profile of voters, the formulation of statements, and the criteria used to define a consensus (Skinner et al. 2015). First, voters were recruited during a General Medicine Congress, which may introduce a selection bias, as participating physicians might be more inclined toward research and scientific advances. Secondly, while the composition of the voting group is diverse and representative of various practice settings in France, it may still be influenced by the personal practices of each expert. Moreover, the formulation of statements was guided by existing literature, which may not fully reflect the diversity of local clinical practices. The implementation of this consensus in daily practice will also depend on the specific context of each physician and patient, as well as the evolution of knowledge and recommendations.

Regarding the statements, a thorough and comprehensive literature review enabled us to identify the main issues encountered in clinical practice and to propose precise and pragmatic guidelines for non‐sleep specialist physicians. As for the threshold used to define consensus, our study followed a rigorous two‐criterion approach: the consensus rate (≥ 75% of responses scoring 7 or higher) to assess overall agreement, and the median (≥ 8) to illustrate response distribution. This strict and demanding definition lends strong credibility to the results. This consensus was built upon the DSM Primary Care (PC) criteria, which evolved to accommodate the diagnostic realities of generalist settings. These criteria adopt accessible thresholds for symptom frequency, duration, and distress, facilitating early identification of insomnia in busy or resource‐limited clinical contexts. As the DSM‐PC criteria are closely aligned with the target population of this study and the routine clinical approach of French general practitioners, this ensured that our recommendations remain grounded in actual practice and applicable to real‐world conditions. Relying on more restrictive or specialist‐driven criteria would have compromised the validity and feasibility of the framework in primary care. Furthermore, this study was conducted with complete and continuous separation between the panel experts and the SC members, who neither participated in voting nor interacted directly with the panellists, ensuring total anonymity.

This Delphi consensus study aims to guide practitioners in their daily practice, while allowing them to remain in control of their prescriptions and tailor them to the patient and circumstances. While this Delphi consensus offers a structured and pragmatic framework to assist general practitioners in managing insomnia complaints, its impact on real‐world clinical outcomes remains to be evaluated. Future studies will be conducted following the online release of the tool in order to assess its real‐world impact, such as its effectiveness in reducing hypnotic use and improving the identification of comorbid sleep disorders.

This consensus complements the 2023 European insomnia guidelines (Riemann et al. 2023) by addressing the management of insomnia specifically in general practice, a setting often underrepresented in specialist guidelines. While the European recommendations provide detailed guidance for specialist contexts, such as sequencing of CBT‐I and detailed pharmacological strategies, they offer limited operationalization for non‐specialist physicians facing real‐world constraints such as limited time, reduced access to CBT‐I, and frequent comorbidities. Our framework specifically addresses this ‘grey zone’ by translating evidence‐based principles into pragmatic, stepwise strategies adapted to generalist contexts. It emphasises initial syndromic assessment, simplified therapeutic pathways, and accessible tools for both diagnosis and follow‐up. By focusing on the practical needs and clinical realities of GPs, our consensus offers a complementary approach designed to enhance applicability, feasibility, and early intervention in frontline care.

## 5 Conclusion

This study provides a foundation for establishing standardised practices in the management of insomnia disorder by frontline practitioners. To our knowledge, it is the first French Delphi consensus designed for non‐sleep specialist physicians to manage insomnia complaints. It outlines a step‐by‐step approach regarding diagnosis, severity assessment, management, specific complementary examinations, and referral to specialists.

Overall, this national Delphi consensus provides practical recommendations for insomnia disorder management, going beyond previous recommendations by offering a pragmatic approach tailored to the practice of frontline physicians.

## Author Contributions

**Pierre‐Alexis Geoffroy:** conceptualization, writing – original draft, methodology, validation, writing – review and editing, resources. **Jean‐Louis Pépin:** conceptualization, writing – original draft, methodology, validation, writing – review and editing, resources. **Marjorie Guillet:** conceptualization, writing – original draft, methodology, validation, writing – review and editing, resources. **Jean‐Bastien Micoulaud‐Franchi:** conceptualization, writing – original draft, methodology, validation, writing – review and editing, resources. **Yves Dauvilliers:** conceptualization, writing – original draft, writing – review and editing, validation, methodology, resources. **Damien Léger:** conceptualization, writing – original draft, methodology, validation, writing – review and editing, resources. **Sylvie Royant‐Parola:** conceptualization, writing – original draft, writing – review and editing, validation, methodology, resources.

## Conflicts of Interest

P.A.G. declares having received funds for seminars, board engagements, consulting, and travel to conferences by Apneal, Biocodex, Bioprojet, Dayvia, Di & Care, Ibsa, Idorsia, Isis Medical, Janssen‐Cilag, Jazz Pharmaceuticals, Lundbeck, Myndblue, MySommeil, Posos, ResilEyes, and Withings. J.L.P. declares having receiving funds for seminars, board engagements and travel to conferences by Idorsia, Takeda, Bioprojet and Pharmaovia. J.L.P. is supported by the French National Research Agency (ANR) in the framework of the “FRANCE 2030” program, the “e‐health and integrated care” chair of Grenoble Alpes University Foundation and “Sleep Health‐AI chair” in “MIAI Cluster” of artificial intelligence (ANR‐23‐IACL‐0006). Y.D. declares having received funds for seminars, board engagements, and travel to conferences by Jazz, Idorsia, Takeda, Avadel, and Bioprojet. D.L. declares having received grants for him or his institution in the past 5 years from Actelion, Sanofi, Vanda, Bioserenity, Vitalaire, Linde. S.R.P. declares having received funds for seminars, board engagements, consulting, and travel to conferences by Asten, Bioprojet, GL Pharma, Idorsia, Linde, Orkyn, Philips, Resmed, SOS Oxygène, Vitalair. The other authors declare no conflicts of interest.

## Supporting information


**Appendix S1.** Application of the Modified Delphi Method.

**Appendix S2.** Characteristics of the General Practitioners in the Voting Group.

**Appendix S3.** Geographical Distribution of Voting General Practitioners.

## Data Availability

The data that support the findings of this study are available from the corresponding author upon reasonable request.
