# Supplementary material for: Management of Insomnia Complaints by Non‐Sleep Specialist Physicians: A French DELPHI Consensus
Source: J Sleep Res. 2025 Jul 17;35(2):e70143. doi: 10.1111/jsr.70143 (PMC13003308; doi:10.1111/jsr.70143)
Supplement: Supplementary file 1 — Appendix S1. Application of the Modified Delphi Method. [file JSR-35-e70143-s001.docx]

**Appendix 1: Application of the Modified Delphi Method**
