# Supplementary material for: Management of Insomnia Complaints by Non‐Sleep Specialist Physicians: A French DELPHI Consensus
Source: J Sleep Res. 2025 Jul 17;35(2):e70143. doi: 10.1111/jsr.70143 (PMC13003308; doi:10.1111/jsr.70143)
Supplement: Supplementary file 2 — Appendix S2. Characteristics of the General Practitioners in the Voting Group. [file JSR-35-e70143-s002.docx]

**Appendix 2: Characteristics of the General Practitioners in the Voting Group**

|  | **Voting Group** (n=37) |
| --- | --- |
| **Median age** in years (range) | 39 (29-63) |
| **Sex** **F/M**: (n) | 70% (26) / 30% (11) |
| **Primary practice setting**: (n)  Private practice  Mixed practice  Hospital-based practice | 73% (27)  19% (7)  8% (3) |
| **Median years of practice** (range) | 10 (1-30) |
| **Average number of consultation days per week** (range) | 4 (2.5-5) |
| **Average number of patients seen per consultation day** (range) | 22 (5-40) |
| **Average number of spontaneous insomnia complaints per week of consultations** (range) | 5 (2-15) |
| **Average number of insomnia complaints expressed after physician inquiry per week of consultations** (range) | 7 (0-20) |
