# Supplementary material for: Management of Insomnia Complaints by Non‐Sleep Specialist Physicians: A French DELPHI Consensus
Source: J Sleep Res. 2025 Jul 17;35(2):e70143. doi: 10.1111/jsr.70143 (PMC13003308; doi:10.1111/jsr.70143)
Supplement: Supplementary file 3 — Appendix S3. Geographical Distribution of Voting General Practitioners. [file JSR-35-e70143-s003.docx]

**Appendix 3: Geographical Distribution of Voting General Practitioners**
